# Simulation of optimal dose regimens of photoactivated curcumin for antimicrobial resistance pneumonia in COVID-19 patients: A modeling approach

**DOI:** 10.1016/j.idm.2023.05.013

**Published:** 2023-06-04

**Authors:** Teerachat Saeheng, Kesara Na-Bangchang

**Affiliations:** a Centre of Excellence in Pharmacology and Molecular Biology of Malaria and Cholangiocarcinoma, Chulabhorn International College, 99 Moo 18, Phaholyothin Road, Thammasat University (Rangsit Campus), Klongneung, Klongluang District, Pathumthani, 12121, Thailand; b Drug Discovery and Development Centre, Office of Advanced Science and Technology, 99 Moo 18, Phaholyothin Road, Thammasat University (Rangsit Campus), Klongneung, Klongluang, Pathumthani, 12121, Thailand

**Keywords:** PBPK, MRSA, VRSA, Photoactivated-curcumin, COVID-19, Pneumonia, Antimicrobial-resistance bacteria, Pharmacokinetics

## Abstract

**Background:**

Secondary antimicrobial resistance bacterial (AMR) pneumonia could lead to an increase in mortality in COVID-19 patients, particularly of geriatric patients with underlying diseases. The comedication of current medicines for AMR pneumonia with corticosteroids may lead to suboptimal treatment or toxicities due to drug-drug interactions (DDIs).

**Objective:**

This study aimed to propose new promising dosage regimens of photoactivated curcumin when co-administered with corticosteroids for the treatment of antimicrobial resistance (AMR) pneumonia in COVID-19 patients.

**Methods:**

A whole-body physiologically-based pharmacokinetic (PBPK) with the simplified lung compartments model was built and verified following standard model verification (absolute average-folding error or AAFEs). The pharmacokinetic properties of photoactivated were assumed to be similar to curcumin due to minor changes in physiochemical properties of compound by photoactivation. The acceptable AAFEs values were within 2-fold. The verified model was used to simulate new regimens for different formulations of photoactivated curcumin.

**Results:**

The AAFEs was 1.12-fold. Original formulation (120 mg once-daily dose) or new intramuscular nano-formulation (100 mg with a release rate of 10/h given every 7 days) is suitable for outpatients with MRSA pneumonia to improve patient adherence. New intravenous formulation (2000 mg twice-daily doses) is for hospitalized patients with both MRSA and VRSA pneumonia.

**Conclusion:**

The PBPK models, in conjunction with MIC and applied physiological changes in COVID-19 patients, is a potential tool to predict optimal dosage regimens of photoactivated curcumin for the treatment of co-infected AMR pneumonia in COVID-19 patients. Each formulation is appropriate for different patient conditions and pathogens.

## Highlights

• New simplified lung PBPK/PD model offers the prediction of desirable drug concentrations at the target sites.
• Precisely predicting appropriate drug concentrations at the target prevents treatment failure and drug resistance.
• This quantitative pharmacology provides potential dosage regimens for antimicrobial resistance therapy in clinical trials. Quantitative pharmacology provides potential dosage regimens for antimicrobial resistance therapy.
• A weekly long-acting nano-formulation is a suitable regimen for out-patients with improving adhrence.

## 1 Introduction

Severe respiratory coronavirus 2 (SARs-CoV-2) infection results in severe complications such as acute respiratory distress syndrome. It is noted that subsequent co-infection with methicillin-resistance *Staphylococcus aureus* (MRSA), vancomycin-resistant *S. aureus* (VRSA), and Candida albicans in coronavirus disease 2019 (COVID-19) leads to severe clinical outcomes and increased mortality rate (Adalbert et al., 2021), particularly in geriatric patients. A recent study in COVID-19 patients showed increasing prevalence of MRSA infection from 0.6% on day 1–5.7% on day 28 (Punjabi et al., 2021). In addition, 57 out of 115 patients (49.6%) were infected with MRSA, and 30 out of 115 patients (26.1%) were co-infected with more than two pathogens (*e.g*., Candida spp.) in COVID-19 patients (Adalbert et al., 2021). The mortality rate was up to 61.7%, indicating the significant contribution of microbial co-infection in COVID-19 patients (Adalbert et al., 2021). The co-infection of VRSA in patients with COVID-19 has not been reported. This pathogen is however, still an important multidrug resistant pathogen that threatens public health. A systematic review and meta-analysis of VRSA prevalence in 2020 reported that the prevalence of VRSA was increasing globally (Wu et al., 2021). The co-infected VRSA in patients with COVID-19 would lead to an increase in mortality. Despite the increase in the prevalence of MRSA and VRSA, treatment options are still limited. Currently used drugs for MRSA and VRSA include linezolid, daptomycin, and iclaprim (Kalimuddin et al., 2014). The use of these drugs for the treatment of bacterial co-infection in patients with COVID-19 is limited due to potential drug-drug interaction (DDIs) with corticosteroids (Data are retrieved from drug bank for drug-drug interactions), resulting in suboptimal treatment or toxicities. Moreover, the resistance of these bacteria to antibiotics has been reported (Morroni et al., 2018; Shariati et al., 2020).^.^ With the limited utility of the current antibiotics for the treatment of MRSA and VRSA infection, the findings of new effective drugs for MRSA and VRSA treatment in patients co-infected with COVID-19 are needed or in the future when the co-administration between corticosteroids and antimicrobial drugs for drug-resistant pathogens are needed.

Curcumin, activated by photoactivation call “photoactivated curcumin”, is a new potential compound to treat antimicrobial resistance (AMR) in pathogens, including MRSA, VRSA, and C. albicans spp., with a low minimum inhibitory concentration (MIC) (Akhtar et al., 2021; Maiolo et al., 2016)^.^ In addition, this compound is unlikely to cause DDIs when co-administered with corticosteroids, as the cytochrome (CYP) P450 is not a major metabolizing enzyme for biotransformation of curcumin. Photoactivated curcumin is, therefore, the preferred drug of choice for AMR pneumonia treatment (MRSA, VRSA, and C. albicans spp.) in COVID-19 patients. However, no optimal dosage regimens for the treatment of MRSA, VRSA, and C. albicans have been recommended.

Physiologically-based pharmacokinetic (PBPK) model has been used to describe drug disposition in the human body by mimicking human physiology, anatomy (dividing human anatomy into compartments), and pathophysiology according to the disease progression (Nestorov, 2003). As a physiologically realistic compartmental structure, the model could predict the drug concentrations at a target site of action. With the advancement of model simulation, virtual populations following Monte Carlo simulation have been applied to simulate the drug concentration-time profiles in the desired populations, which could assist in finding the different dosage regimens (Nestorov, 2003). A whole-PBPK model is, therefore, the potential tool in predicting optimal dosage regimens in infective diseases (Rayner et al., 2021).

The aim of this study was to determine the promising dosage regimens of photoactivated curcumin when co-administered with corticosteroids for the treatment of AMR pneumonia in COVID-19 patients.

## 2 Materials and methods

### 2.1 Model construction

**Fig. 1:**
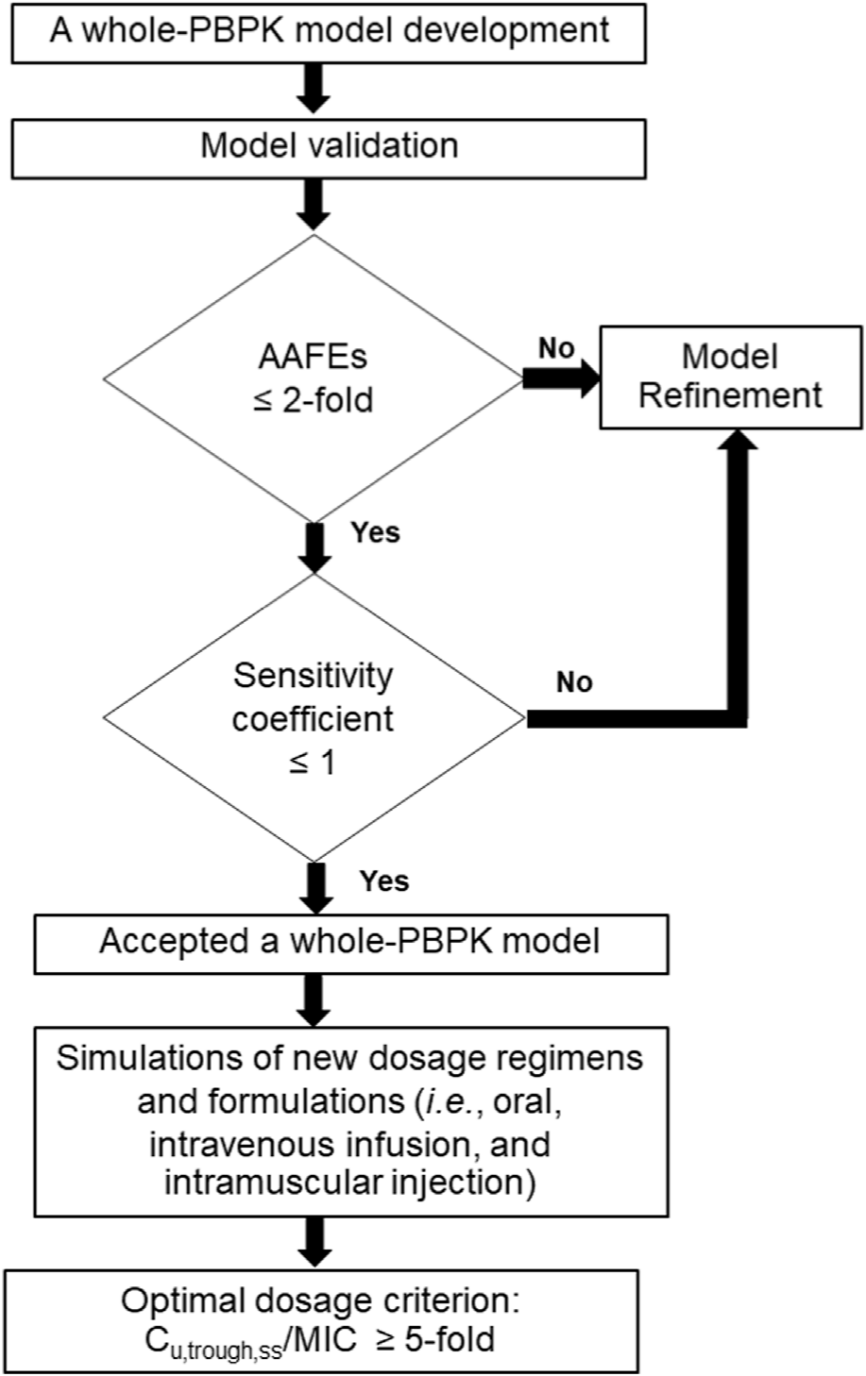
A flowchart diagram of the prediction of suggested dosage regimens.

The whole-body PBPK models (curcumin, rifampicin, and lopinavir/ritonavir) were built using Simbiology® (a product of MATLAB, version 2018a, MathWorks, Natick, MA., USA) based on the previously published articles (Rajoli et al., 2015; Saeheng et al., 2019). It is noted that The model parameters are shown in Supplemental Table 1. Model assumptions were blood flow-limited organs, except for the lungs compartment (permeability-limited model), rapid distribution of compound into the tissue, similarity of pharmacokinetic properties between curcumin and photoactivated curcumin, and absence of absorption at the large intestine and entero-hepatic recirculation. One hundred virtual populations (aged 18–60 years, 50 male and 50 female, average weighting 70 kgs) were simulated using Monte Carlo simulation were performed for all dosage regimens and formulations. A flowchart diagram for dose optimization is shown in Fig. 1.

### 2.2 Model verification

The predicted and observed results of curcumin, rifampicin, and lopinavir were compared. The average absolute-folding errors (AAFEs), a standard for model verification, were calculated to determine the prediction accuracy. The AAFEs equation is shown below:

(1) $$AAFEs=10^{\sum_{i=1,2,3,..,n}^{n}\frac{\left|\log\frac{prediction}{observation}\right|}{n}}$$

AAFEs is the average absolute-folding errors; n is the number of model parameters; prediction is the simulated result; observation is the observed result. The acceptable range for AAFEs is within 2-fold (Saeheng et al., 2018).

### 2.3 Sensitivity analysis

The sensitivity coefficient for selected model parameters was calculated to determine the effect of model parameters on the changes in plasma concentrations, which indicates model certainty. For photoactivated curcumin, the following parameters, *i.e*., the fraction of unbound drug (f_u_), absorption rate (K_a_), liver weight, intestinal weight, blood-to-plasma partition ratio (R_bp_), blood flow to the gut (Q_gut_), hepatic portal vein blood flow (Q_hv_), and water-solubility were selected for determination of the effects of these model parameters on the changes of plasma concentration. The values were varied by ±20% from mean values. The sensitivity analysis of the development of a new lung compartment was performed following a single dose of 600 mg rifampicin. These sensitivity coefficients were used to determine the effects of model parameters on the drug concentration profiles in bronchial mucosa (BM) and epithelial lining fluid (ELF). The model parameters were f_u,t_, apparent permeability from basal to apical (P_app_, _B-to-A_), apparent permeability from apical to basal (P_app, A-to-B_) pH of ELF, and pH of BM. The equation is shown below:

(2) Φ _I_ = %Δy/%ΔX

Φ I is the sensitivity coefficient; %Δy and %ΔX are the percent changes of curcumin plasma concentration and the model parameter, respectively.

### 2.4 Minimal inhibitory concentration (MIC)

Three antimicrobial resistance pneumonia infections included in the study were MRSA ATCC 43300, VRSA, and C. albicans ATCC 90028. The MIC values of these three pathogens were 0.25, 156, and 2 mg/L, respectively (Akhtar et al., 2021; Maiolo et al., 2016). The ratios of the simulated unbound trough concentration at steady-state in the bronchial mucosa (BM), including MIC (C_ut, trough, ss_/MIC) were calculated to determine the folding differences between C_u, trough, ss_ and MIC. Cu, trough, ss/MIC ratios are presented as mean ± 95% confidence interval (CI) values. The total simulation time was 21 days based on the duration of antibiotic treatment for community acquired pneumonia (CAP) (Pinzone et al., 2014).

### 2.5 Impact of physiological changes in COVID-19 patients

The epithelial lining fluid (ELF) pH is acidic acid, ranging from 6.9 (5.7–7.5) in healthy subjects (Zajac et al., 2021). However, the ELF pH in patients with bacterial pneumonia was decreased to 5.6 (Rowland-Yeo et al., 2020). To our knowledge, the ELF pH values in viral pneumonia infection and in COVID-19 patients are not available. The ELF pH in this study was therefore set as 5.6. Besides, the ELF pH changes in patients with pneumonia, serum albumin and alpha-1-acid glycoprotein (AAG) concentrations were also altered. The average serum albumin in patients with viral pneumonia was decreased to 29.3 g/L (Rowland-Yeo et al., 2020), which was similar to COVID-19 patients (31 g/L) (Salinas et al., 2021). The alteration of serum albumin levels directly results in changes in f_u_. However, based on its pKa value, photoactivated curcumin is a basic drug that binds to AAG rather than albumin Such changes in serum albumin levels in COVID-19 patients are unlikely to be influenced by its f_u_. AAG levels in COVID-19 patients were increased by 2-fold compared with healthy subjects (Rowland-Yeo et al., 2020). Since the altered AAG levels also influence f_u_, the effects of 2-fold changes of f_u_ on C_u_, _trough, ss_ in bronchial mucosa were determined using the sensitivity analysis. In addition, the median estimated glomerular filtration (eGFR) in COVID-19 patients is decreased to 39 mL/min/1.73 m^2^, resulting in decrease in renal clearance (Taylor et al., 2021). Nevertheless, the renal clearance of photoactivated curcumin is less than 10% (Sharma et al., 2004). Such an effect of a decrease in renal clearance is likely to be neglectable.

### 2.6 Simulation of oral dose regimens

Since the maximal dosage regimen in healthy subjects was 12,000 mg (Cheng et al., 2001), the initial simulated dose regimen was 3000 mg given orally four times daily. The simulated dose reduction was performed until the C_u, trough, ss_/MIC ratios were lower than one. In case when none of the current formulations could provide the C_u, trough, ss_/MIC ratios for the VRSA pneumonia treatment, the new oral formulation dosage regimens were simulated with a fixed increase of 20-fold of apparent permeability (P_app_) and the fixed increase of 10-fold of water-solubility.

### 2.7 Simulation of intravenous infusion dose regimens

The intravenous infusion depot was created and given directly to the venial compartment. The infusion rate for intravenous infusion is shown below:

(3) Infusion rate = (amount of given dose)/(period of the given dose)

Since the maximal safety dosage regimen for intravenous infusion was 250 mg given as once-daily dose (Storka et al., 2015), the initial simulated dosage regimen was based on this dosage regimen in healthy subjects. In case when the maximal dosage regimen did not provide adequate C_u, trough, ss_/MIC ratios for VRSA pneumonia treatment, the new intravenous formulation was simulated. The maximal dosage for intravenous infusion was the half-maximal dosage of the oral dose (6000 mg) (Cheng et al., 2001).

### 2.8 Simulation of intramuscular long-acting injectable nano-formulation (LAI) dose regimens

The intramuscular injection depot was created based on a previously published article (Rajoli et al., 2015) The maximal dosage and maximal release rate (K_im_) were 1500 mg in 6 mL, and 0.015/h (Rajoli et al., 2015), respectively. The K_im_ was increased for the new formulation when the current Kim did not provide adequate C_u, trough,_ _ss_ until Cu, trough, ss/MIC ratios (>1).

### 2.9 Criteria for optimal dosage regimen

C_u, trough, ss_ prediction at target site based on PBPK modeling represents the dynamic of the drug concentrations explicitly killing effect of the drug to pathogens better than traditional PK/PD indices**.** This issue would be recognized as the new concept of model-informed drug discovery and development (MIDD) in current Prescription Drug User Fee Act (PDUFA) (Wang et al., 2019).

As a result, the C_u, trough, ss_/MIC ratios at target site would be a surrogate of optimal dosage regimen for antimicrobial therapy. C_u, trough, ss/_MIC ratios have limited to many pathogens. To our knowledge, only the susceptibility test of *S. aureus* on vancomycin using C_u, trough, ss_/MIC over 4-fold in various classes of antibiotics with the bacterial killing effect ranging from 90% to 99.9% was published (Bozdogan et al., 2003). The suggested dosage regimens for antimicrobial therapy with different formulations were determined based on the C_u, trough, ss/_MIC ratios at least 5-fold.

## 3 Results

### 3.1 Model verification and sensitivity analysis

For curcumin, the AAFEs for predicted dosage regimens (i.e., 4,000, 6,000, and 8000 mg) was 1.12-fold. The corresponding values for 4,000, 6,000, and 8000 mg were 1.22, 1.08, and 1.07-fold, respectively (Cheng et al., 2001). For rifampicin and lopinavir, AAFEs of ELF and bronchial mucosa (rifampicin) and ELF were 1.09 (1.02–1.17) (Atzori et al., 2003; Eron et al., 2004; Rafiq et al., 2010; Rasool et al., 2019; Ziglam et al., 2002), and 1.19 (Ziglam et al., 2002), respectively. In addition, the AAFEs for lopinavir and rifampicin in plasma were 1.18 (1.08–1.27) (Atzori et al., 2003; Eron et al., 2004; Rafiq et al., 2010), and 1.16 (1.13–1.18) (Rafiq et al., 2010; Rasool et al., 2019), respectively. The virtual predictive checks (VPCs) for each dose are shown in Supplemental Figs. 1–3, respectively. For photoactivated curcumin, the sensitivity coefficients for f_u_, K_a_, liver weight, intestinal weight, R_bp_, Q_gut_, Q_hv_, and water-solubility were −0.56, −0.6, −0.08, −0.07, 0, +0.01, +0.87, and +0.97, respectively. The sensitivity coefficients (ELF) for f _u, t_, pH of BM, pH of ELF, P_app, A-to-B_, and P_app, B-to-A_ were −0.53, −0.16, −0.05, −0.07, −0.60, respectively. The values for BM were +0.56, −0.18, −0.08, −0.102, and −0.61, respectively. The effect of the 2-fold change of fu on C_u, trough, ss_ in bronchial mucosa was determined using sensitivity coefficient analysis. The sensitivity coefficient was −0.04, indicating no effects of changes of fu on C_u, trough, ss_ in bronchial mucosa. Therefore, the fu was not changed, while the AAG level was increased during COVID-19 infection. A positive sensitivity coefficient suggests an increase in a model parameter that results in an increase in plasma concentration, while the negative sensitivity coefficient suggests an increase in a model parameter that results in a decrease in plasma concentration.

### 3.2 Simulation of oral dose regimens

Current oral formulation: For four-times daily doses, all regimens provided the C_u, trough, ss_/MIC ratios greater than one, ranging from 7.88 to 25.69 for MRSA ATCC 433000, and from 63 to 205 for *C. albicans* ATCC 90028 (Table 1). For twice-daily doses, the corresponding values following regimen-IG to IJ ranged from 1.66 to 7.40, and from 13.25 to 59.20, respectively (Table 1). For once-daily doses, these values for MRSA ATCC 43300 following regimen-IK were higher than regimen-IL (Table 1). Notably, the C_u, trough, ss/_MIC ratios for *C. albicans* ATCC 90028 following regimen-IL, but not for regimen-IK were lower than one (Table 1). None of the current oral formulations provided the C_u, trough, ss_/MIC ratios for VRSA greater than one (Table 1).

**Table 1:** The C_u, trough and ss_/MIC ratios for current oral formulation with different dosage regimens. Data are presented as mean ± (95% confidence interval).

Regimen	MRSA ATCC 43300	VRSA	*C albicans* ATCC 90028
3000 mg QID (regimen-IA)	205.56 (188.9–222.23)	0.33 (0.30–0.35)	25.69 (23.6–27.79)
2000 mg QID (regimen-IB)	193.26 (179.08–207.08)	0.31 (0.29–0.33)	24.16 (22.39–25.93)
1000 mg QID (regimen-IC)	172.73 (160.97–184.49)	0.27 (0.26–0.29)	21.59 (20.11–23.06)
500 mg QID (regimen-ID)	144.66 (136.03–153.28)	0.23 (0.21–0.24)	18.02 (17.00–19.00)
250 mg QID (regimen-IE)	111.00 (102.87–119.14)	0.177 (0.164–0.190)	13.87 (12.86–14.89)
125 mg QID (regimen-IF)	63.09 (59.29–66.89)	0.11 (0.09–0.11)	7.88 (7.41–8.36)
500 mg BID (regimen-IG)	59.20 (55.44–62.95)	0.09 (0.08–0.10)	7.40 (6.90–7.86)
250 mg BID (regimen-IH)	45.73 (41.92–49.54)	0.07 (0.06–0.08)	5.73 (5.24–6.19)
125 mg BID (regimen-II)	26.83 (24.70–28.96)	0.043 (0.039–0.046)	3.55 (3.08–3.62)
60 mg BID (regimen-IJ)	13.25 (12.23–14.27)	0.021 (0.019–0.022)	1.66 (1.53–1.78)
120 mg OD (regimen-IK)	8.72 (7.81–9.63)	0.013 (0.012–0.015)	1.09 (0.97–1.20)
60 mg OD (regimen-IL)	4.55 (4.04–5.05)	0.007 (0.006–0.008)	0.57 (0.50–0.63)

QID: four times a day; BID: two times a day; OD: once time a day.

New oral formulation: For four-times daily doses, the highest C_u, trough, ss_/MIC ratios were observed following regimen-IIA for MRSA ATCC 43300, VRSA and C. albicans ATCC 90028 compared with other regimens (Table 2). With the lowest dosage regimen (regimen-IIC) of four-time daily doses, the corresponding values were still higher than 4-fold for all pathogens, ranging from 4.41 to 2752 (Table 2). For the three-times daily doses, the C_u, trough, ss_/MIC ratios following all regimens for MRSA ATCC 43300, VRSA and *C. albicans* ATCC 90028 were greater than one. The highest C_u, trough, ss_/MIC ratios were observed following regimen-IID (highest dosage regimen) for MRSA ATCC 43300, VRSA and *C. albicans* ATCC 90028 compared with the others (Table 2). The corresponding values following dose of the lowest amount of dosage administration (regimen-IIH) ranged from 1.64 to 1027. For the twice-daily doses, the values following regimen-III (highest dosage for twice-daily doses) were comparable with the highest dose regimen of four-times daily (regimen-IIA). With the lowest dose regimen (regimen-IIL), the corresponding values for MRSA ATCC 43300, VRSA, and *C. albicans* ATCC 90028 were greater than one, ranging from 1.95 to 1216 (Table 2). The simulated results for all dosage regimens are shown in Table 2.

**Table 2:** The C_u, trough and ss_/MIC ratios for new oral formulation with different dosage regimens. Data are presented as mean ± (95% confidence interval).

Regimen	MRSA ATCC 43300	VRSA	*C albicans* ATCC 90028
3000 mg QID (regimen-IIA)	7795 (7256–8335)	12.49 (11.62–13.36)	974 (907–1041)
2000 mg QID (regimen-IIB)	5504 (5157–5851)	8.82 (8.27–9.37)	688 (646–729)
1000 mg QID (regimen-IIC)	2752 (2564–2941)	4.41 (4.10–4.71)	344 (320–367)
4000 mg TID (regimen-IID)	8370 (7898–8842)	13.41 (12.65–14.17)	1046 (987–1105)
3000 mg TID (regimen-IIE)	6085 (5684–6486)	9.75 (9.10–10.39)	760 (710–810)
2000 mg TID (regimen-IIF)	4013 (3747–4279)	6.43 (6.01–6.84)	501 (469–533)
1000 mg TID (regimen-IIG)	2071 (1940–2203)	3.32 (3.11–3.53)	258 (242–275)
500 mg TID (regimen-IIH)	1027 (956–1097)	1.64 (1.53–1.75)	128 (119–137)
6000 mg BID (regimen-III)	7431 (7009–7853)	11.91 (11.23–12.56)	929 (876–981)
3000 mg BID (regimen-IIJ)	3712 (3452–3972)	5.94 (5.53–6.37)	464 (431–496)
2000 mg BID (regimen-IIK)	2512 (2347–2678)	4.02 (3.76–4.29)	314 (293–334)
1000 mg BID (regimen-IIL)	1216 (1126–1306)	1.95 (1.80–2.09)	152 (140–163)
125 mg OD (regimen-IIM)	61.5 (56.48–66.52)	0.098 (0.09–0.106)	7.68 (7.06–8.31)
25 mg OD (regimen-IIN)	11.66 (10.85–12.47)	0.018 (0.017–0.019)	1.46 (1.36–1.56)

QID: four times a day; TID: three time a day; BID: two times a day.

### 3.3 Simulation of intravenous dose regimens

Current intravenous formulation: For the once-time daily dose, the Cu, trough, ss/MIC ratios following the maximal dosage administration (regimen-IIIA; 250 mg) for MRSA ATCC 43300, VRSA, and *C. albicans* ATCC 90028 were highest compared with the other regimens (0.35–219). The corresponding values following the lowest amount of dosage (regimen-IIID) ranged from 0.026 to 16.37 (Table 3). It was noted that the C_u, trough, ss_/MIC ratio for VRSA following current intravenous formulation was lower than one. All results are shown in Table 3.

**Table 3:** The _Cu, trough and ss_/MIC ratios for current intravenous formulation with different dosage regimens. Data are presented as mean ± (95% confidence interval).

Regimen	MRSA ATCC 43300	VRSA	*C albicans* ATCC 90028
250 mg IV infusion over 24 h q24 h (regimen-IIIA)	219 (205–234)	0.35 (0.32–0.37)	27.48 (25.67–29.30)
125 mg IV infusion over 24 h q24 h (regimen-IIIB)	102.80 (95.70–109.90)	0.16 (0.15–0.17)	12.85 (11.97–13.73)
75 mg IV infusion over 24 h q24 h (regimen-IIIC)	57.11 (53.35–60.85)	0.09 (0.08–0.10)	7.13 (6.67–7.61)
50 mg IV infusion over 24 h q24 h (regimen-IIID)	49.33 (46.19–52.47)	0.08 (0.07–0.08)	6.17 (5.77–6.55)
25 mg IV infusion over 24 h q24 h (regimen-IIIE)	16.37 (15.43–17.31)	0.026 (0.025–0.028)	2.05 (1.93–2.16)

IV: intravenous.

New intravenous formulation: For twice-daily doses, the highest C_u, trough, ss/_MIC ratios (8.86–5532) were observed following the regimen-IVA (3000 mg) for MRSA ATCC 43300, VRSA, and *C. albicans* ATCC 90028 compared with other regimens (Table 4). When the amount of dose was decreased to 250 mg (regimen-IVE), the values were higher than one for MRSA ATCC 43300 and *C. albicans* ATCC 90028, but not for VRSA (Table 4). In addition, the lowest regimen (regimen-IVD, 500 mg) provided the C_u, trough, ss_/MIC ratios greater than one for all pathogens (Table 4).

**Table 4:** The C_u and trough, ss_/MIC ratios for new intravenous formulations with different dosage regimens. Data are presented as mean ± (95% confidence interval).

Regimen	MRSA ATCC 43300	VRSA	*C albicans* ATCC 90028
3000 mg IV infusion over 12 h q12 h (regimen-IVA)	5532 (5186–5877)	8.86 (8.33–9.42)	691 (648–734)
2000 mg IV infusion over 12 h q12 h (regimen-IVB)	3732 (3512–3952)	5.98 (5.62–6.33)	466 (439–394)
1000 mg IV infusion over 12 h q12 h (regimen-IVC)	1803 (1683–1923)	2.89 (2.69–3.08)	225 (210–240)
500 mg IV infusion over 12 h q12 h (regimen-IVD)	934 (873–996)	1.49 (1.40–1.60)	116 (109–124)
250 mg IV infusion over 12 h q12 h (regimen-IVE)	471 (448–494)	0.75 (0.72–0.79)	59.00 (56.00–61.00)

IV: intravenous.

### 3.4 Simulation of LAI intramuscular dose regimens

Current LAI nano-formulation: Over all, the current intramuscular formulation (regimen-VA) could not provide sufficient Cu, trough, ss/MIC ratios for all pathogens. The new intramuscular formulations were therefore simulated.

New LAI nano-formulation: For the intramuscular injection given every 7 days, the C_u, trough, ss_/MIC ratios following regimen-VD were the highest (0.14–85.97) compared with the other regimens. The corresponding values for MRSA ATCC43300 and *C. albicans* ATCC90028 following the lowest dose regimen (regimen-VD; 100 mg) ranged from 1.60 to 12.77. It was noted that the C_u, trough, ss_/MIC ratio following the intramuscular injection formulation given every 7 days for VRSA were higher than one. In addition, the C_u, trough, ss_/MIC ratios following the fixed dose of 1500 mg with K_im_ of 10/h (regimen-VH, regimen-VI, and regimen-VJ) for MRSA ATCC43300, and *C. albicans* ATCC 90028 ranged from 56.39 to 1411. It was noted that the values for following regimen-VI and VJ except for regimen-VH were higher than one (1.14–2.26). All simulated C_u, trough, ss_/MIC ratios for all regimens are shown in Table 5.

**Table 5:** The C_u, trough, ss_/MIC ratios for new intramuscular formulation with different dosage regimens. Data are presented as mean ± (95% confidence interval).

Regimen	MRSA ATCC 43300	VRSA	*C albicans* ATCC 90028
1500 mg 0.015 of K_im_ q 7 days (regimen-VA)	0.27 (0.26–0.28)	0.00043 (0.00041–0.00046)	0.034 (0.032–0.035)
1500 mg 0.15 of K_im_ q 7 days (regimen-VB)	3.42 (3.20–3.64)	0.0055 (0.0051–0.0048)	0.43 (0.40–0.45)
1500 mg 1.5 of K_im_ q 7 days (regimen-VC)	35.25 (33.20–37.30)	0.056 (0.053–0.059)	4.41 (4.15–4.66)
1500 mg 10 of K_im_ q 7 days (regimen-VD)	85.97 (76.34–95.55)	0.14 (0.12–0.15)	10.74 (9.53–11.96)
750 mg 5 of K_im_ q 7 days (regime -VE)	46.46 (41.76–51.17)	0.07 (0.06–0.08)	5.80 (5.21–6.40)
375 mg 2.5 of K_im_ q 7 days (regimen-VF)	24.47 (21.65–27.29)	0.04 (0.035–0.043)	3.05 (2.70–3.41)
100 mg 0.6 of K_im_ q 7 days (regimen-VG)	12.77 (12.13–13.42)	0.02 (0.019–0.021)	1.60 (1.51–1.68)
1500 mg 10 of K_im_ q 3 days (regimen-VH)	451 (422–479)	0.72 (0.67–0.77)	56.39 (52.81–59.97)
1500 mg 10 of K_im_ q 2 days (regimen-VI)	713 (671–755)	1.14 (1.07–1.21)	89.18 (83.92–94.47)
1500 mg 10 of K_im_ q24 h (regimen-VJ)	1411 (1328–1494)	2.26 (2.13–2.39)	176 (166–186)

K_im_: intramuscular injection release rate.

**Fig. 2:**
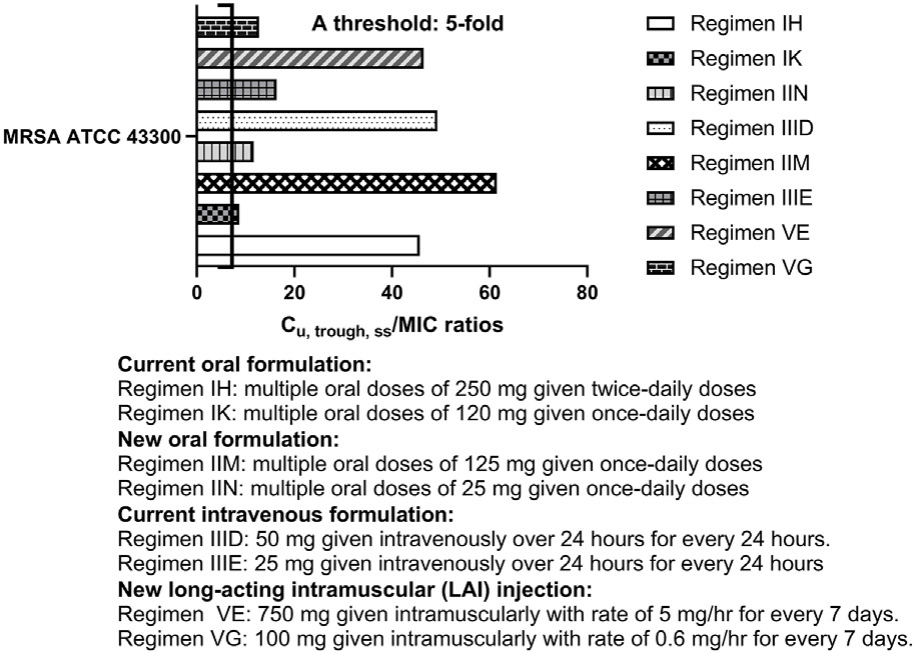
Comparisons of suggested dosage regimens for *MRSA ATCC 43300* therapy.

**Fig. 3:**
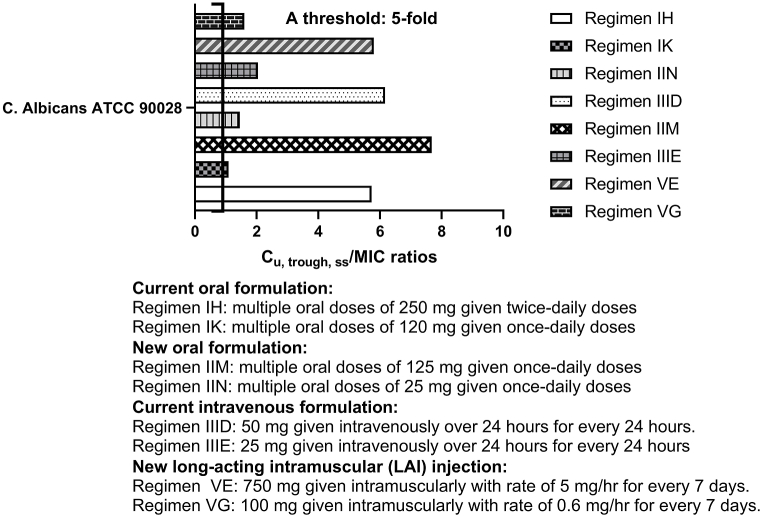
Comparisons of suggested dosage regimens for *C. albicans ATCC 90028* therapy.

**Fig. 4:**
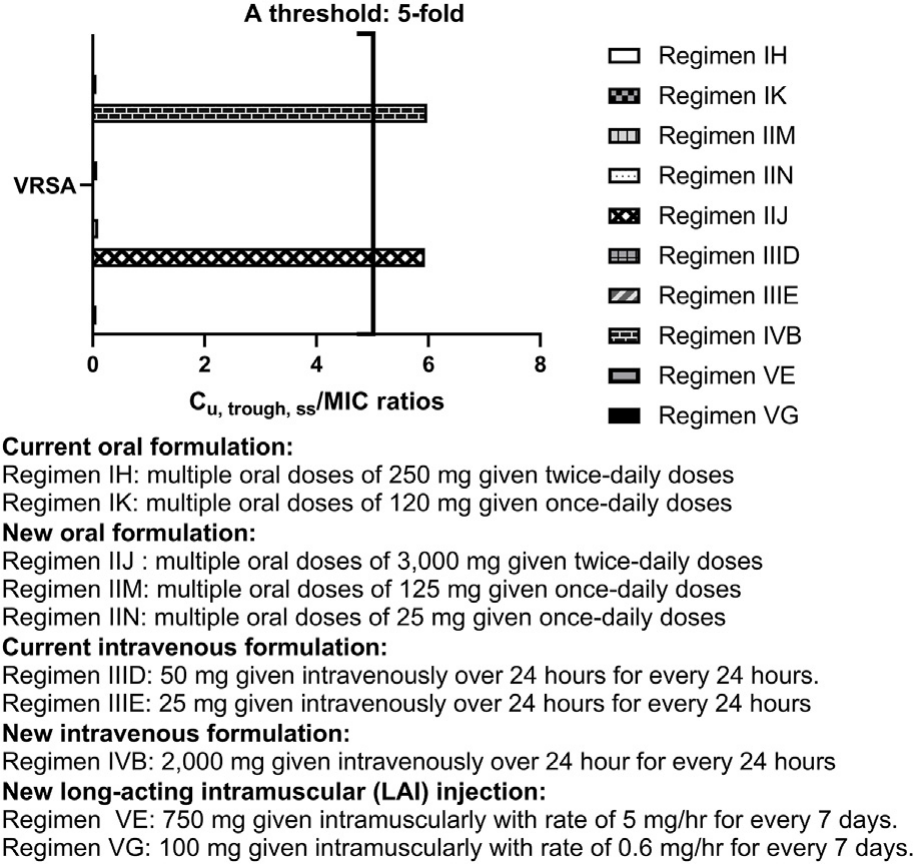
Comparisons of suggested dosage regimens for VRSA therapy.

Comparisons of suggested dosage regimens for MRSA ATCC43300, C. albicans ATCC 90028, and VRSA therapy are shown in Fig. 2, Fig. 3, Fig. 4, respectively.

## 4 Discussion

The PBPK model was successfully developed for predicting curcumin disposition for all dosage regimens. All sensitivity coefficients (sensitivity analysis) were less than one, indicating the insensitivity of model parameters to the plasma concentration. The verified PBPK model is, therefore, credible. The verified PBPK model in conjunction with MIC was applied for predicting potential dosage regimens of photoactivated curcumin co-administration with corticosteroids for the treatment of AMR pneumonia (MRSA, VRSA, and C. albicans) in COVID-19 patients in various formulations.

### 4.1 Simulation of oral dose regimens

Current oral formulation: For the treatment of MRSA ATCC 43300, and *C. albicans* ATCC 90028 pneumonia, regimen-IA showed the best clinical efficacy due to it exhibited the highest C_u, trough, ss_/MIC ratios (Table 1). However, C_u, trough, ss_/MIC ratios, amount of dosage and frequency of drug administration (four-times daily) were too excessive, which may lead to poor patient's adherence to medication, and, thus, treatment failure. The other dosage regimens were optimal. For the treatment of MRSA ATCC 43300 pneumonia, regimen-IK (once-daily dose) was recommended since it provided adequate C_u, trough, ss_/MIC ratios and could improve the patient's adherence In case when the amount of dosage administration was a great concern, regimen-IL was preferable. Nevertheless, for successful treatments of MRSA and VRSA pneumonia, the unbound drug concentrations at the target site should be above the MIC by at least 5-fold for VRSA (Bozdogan et al., 2003). Regimen-IK was, therefore, recommended for the treatment of MRSA ATCC 43300 pneumonia. For the treatment of *C. albicans* ATCC 90028 pneumonia, regimen-IH rather than regimen-IK was recommended due to the inadequate C_u, trough, ss_/MIC ratios.

New oral formulation: For the treatment of MRSA ATCC 43300 pneumonia, the recommended regimen was regimen-IIM. This regimen provided sufficient C_u, trough, ss_/MIC ratios (11-fold) with the lowest dose (25 mg OD) and frequency of drug administration (once-daily dose). For the treatment of *C. albicans* ATCC 90028 pneumonia, regimen-IIN (125 mg OD) was recommended (C_u, trough, ss_/MIC ratios > 5-fold). For the treatment of VRSA pneumonia, regimen-IIJ was suitable. In addition, this regimen also provided sufficient C_u, trough, ss_/MIC ratios for the treatment of three pathogens.

### 4.2 Simulation of intravenous dose regimens

Current intravenous formulation: For the treatment of MRSA ATCC 43300, and *C. albicans* ATCC 90028 pneumonia, regimen-IIID was the best option since it provided sufficient C_u, trough, ss_/MIC ratios. In case when the MRSA ATCC 43300 pneumonia was a great concern, regimen-IIIE was recommended.

New intravenous formulation: For the treatment of VRSA pneumonia, the suitable regimen was regimen-IVB (C_u, trough, ss_/MIC ratios >5). This regimen also covered the treatment of MRSA ATCC 43300 and C. albicans ATCC 90028 pneumonia, but it exhibited extremely high values of C_u, trough, ss_/MIC ratio. The current intravenous formulation (regimen-IIID) was also recommended for the treatment MRSA ATCC 43300, and *C. albicans* ATCC 90028 pneumonia.

### 4.3 Simulation of LAI intramuscular dose regimens

New LAI nano-formulation: For the treatment of MRSA ATCC 43300 pneumonia, regimen-VG was recommended (C_u, trough, ss_/MIC ratios >5). In case when the C. albicans ATCC 90028 and MRSA ATCC 43300 were the targeted pathogens, regimen-VE was recommended since it provided adequate C_u, trough, ss_/MIC ratios for both pathogens. It was noted that none of the intramuscular regimens could provide sufficient Ctrough, ss/MIC ratios (>5) for VRSA pneumonia even with the highest dose (dose of 1500 mg, every 24 h (regimen-VJ)). Accordingly, an intramuscular formulation was not suitable for the treatment of VRSA pneumonia. Besides the treatment in COVID-19 patients, The LA formulation is suitable for out-patients in terms of improve patients’ adherence compared with orally for a long-term therapy.

### 4.4 The co-administration of corticosteroids for the treatment of co-infected AMR pneumonia

Clinical management of bacterial pneumonia in COVID-19 requires the comedications of antibiotics and corticosteroids. Linezolid, daptomycin, telavancin, oritavancin, tigecycline, dalbavancin, ceftobiprole and iclaprim are the currently used antibiotic drugs for MRSA and VRSA. However, linezolid, daptomycin and iclaprim have potential DDIs with corticosteroids (moderate-to-major DDIs). Comedications with these drugs are prone to cause severe adverse effects and increased mortality. Daptomycin, telavancin, oritavancin and iclaprim are excreted by the renal route, and the decrease in eGFR in COVID-19 patients could lead to drug accumulations and toxicities. Besides the DDIs problem, adequate trough, ss/MIC ratios in the bronchial mucosa are the crucial contributing factor to the successful treatment of AMR pneumonia. The C_u, trough, ss_/MIC ratios of MRSA for linezolid, dalbavancin and iclaprim were 2.02 (Buerger et al., 2006; Gotfried et al., 2017; Honeybourne et al., 2003; Xu et al., 2021), 2.5 (Bongiorno et al., 2020; Carrothers et al., 2020), and 0.13 (Andrews et al., 2007), respectively. In addition, the values for MDR, VRSA for dalbavancin and iclaprim were 0.08 (Carrothers et al., 2020; Huang et al., 2017), and 0.008 (Andrews et al., 2007; Huang et al., 2017), respectively. These ratios were calculated based on the available information in citations. The lower 5-fold of C_u, trough, ss_/MIC ratios may lead to treatment failure due to insufficient drug concentration profiles at the target site. These results were in accordance with the low volume of distribution of these drugs (except iclaprim). Altogether with DDIs issue and inadequate C_u, trough, ss_/MIC ratios in bronchial mucosa (site of infection), the current medicines are unlikely to be appropriate for AMR pneumonia in COVID-19 patients. On the other hand, the co-medication of photoactivated curcumin and corticosteroids for the treatment of bacterial pneumonia superinfection is a preferable choice since the metabolizing enzymes involved in biotransformation of photoactivated curcumin are different from that of corticosteroid or molnupiravir or remdesivir. Therefore, the DDIs between photoactivated curcumin and corticosteroids/molnupiravir/remdesivir are unlikely and photoactivated curcumin should be effective for the treatment of AMR pneumonia in COVID-19 patients.

### 4.5 Limitations

The elimination of photoactivated curcumin through biliary and renal excretion, as well as the transport-mediated photoactivated curcumin diffusion did not include in this study due to its negligible amounts excreted in urine and feces (<10%) (Sharma et al., 2004). Photoactivation has less effect on the physicochemical properties of the compound, resulting in minor changes of its properties, which are unlikely to affect its pharmacokinetic properties (i.e., phenothiazine) (Vara et al., 2019). The models were developed based on the similarity in physicochemical and pharmacokinetic properties between curcumin and photoactivated curcumin. Photoactivated curcumin or curcumin concentration profiles in bronchial mucosa and epithelial lining fluid are not available, and the validation of curcumin as the new lung model development is limited. However, lopinavir and rifampicin, were used as surrogates for the validation of the new development of the lung compartment. As a result, this model is credible. In addition, the new lung compartment PBPK models used P_app, A-to-B_ and P_app, B-to-A_ to predict drug concentrations in ELF and bronchial epithelial cell (BEC), this new lung PBPK model could be applied to predict other new drugs for the lung infections.

## 5 Conclusions

In conclusion, the results of the study demonstrate that PBPK model, in conjunction with MIC and applied physiological changes in COVID-19 patients, is a potential tool to predict optimal dosage regimens of photoactivated curcumin for the treatment of co-infected AMR pneumonia in COVID-19 patients. The current oral formulation is more comfortable for non-severe COVID-19 patients (home isolation), while the intravenous formulation is appropriate for hospitalized patients. In case when the patient's adherence is a great concern due to long-term therapy (up to 21 days), the intramuscular formulation is recommended for outpatients since the frequency of administration is only once a week**.**

## CRediT authorship contribution statement

Teerachat Saeheng: Data curation, Formal analysis, Software visualization, Writing original draft; Kesara Na-Bangchang: Conceptualization, Data curation, Writing-review &editing.

## Declaration of competing interest

The authors declare that they have no known competing financial interests or personal relationships that could have appeared to influence the work reported in this paper.

## Abbreviations

AAFEs: Absolute average-folding errors
AAG: alpha-1-acid glycoprotein
AMR: Antimicrobial resistance
COVID-19: Coronavirus disease 2019
CYP450: Cytochrome P450
C_u, trough, ss_: Trough concentration of unbound drug at steady state
DDIs: Drug-drug interactions
eGFR: Estimated glomerular filtration
ELF: Epithelial lining fluid
F_u_: Fraction of unbound drug in plasma
F_u,t_: Fraction of unbound drug in tissue
K_a_: Absorption rate
K_im_: Intramuscular injection release rate
MIC: Minimal inhibitory concentration
MRSA: Methicillin-resistant Staphylococcus Aureus
P_app. A-to-B_: Apparent permeability from apical to basolateral
P_app, B-to-A_: _Apparent_ permeability from basolateral to apical
PBPK: Physiologically-based pharmacokinetic
Q_gut_: Blood flow at gut
Q_hv_: Blood flow at hepatic vein
R_bp_: Blood-to-plasma partition
SARs-CoV-2: Severe respiratory coronavirus-2
