# Supplementary material for: Simulation of optimal dose regimens of photoactivated curcumin for antimicrobial resistance pneumonia in COVID-19 patients: A modeling approach
Source: Infect Dis Model. 2023 Jun 4;8(3):783–93. doi: 10.1016/j.idm.2023.05.013 (PMC10239661; doi:10.1016/j.idm.2023.05.013)
Supplement: Multimedia component 1 [file mmc1.docx]

**Supplementary file**

**Figure S1.** A visualization of serum curcumin concentrations between predicted data and observed data at an oral single dose of 4000 mg curcumin administration.


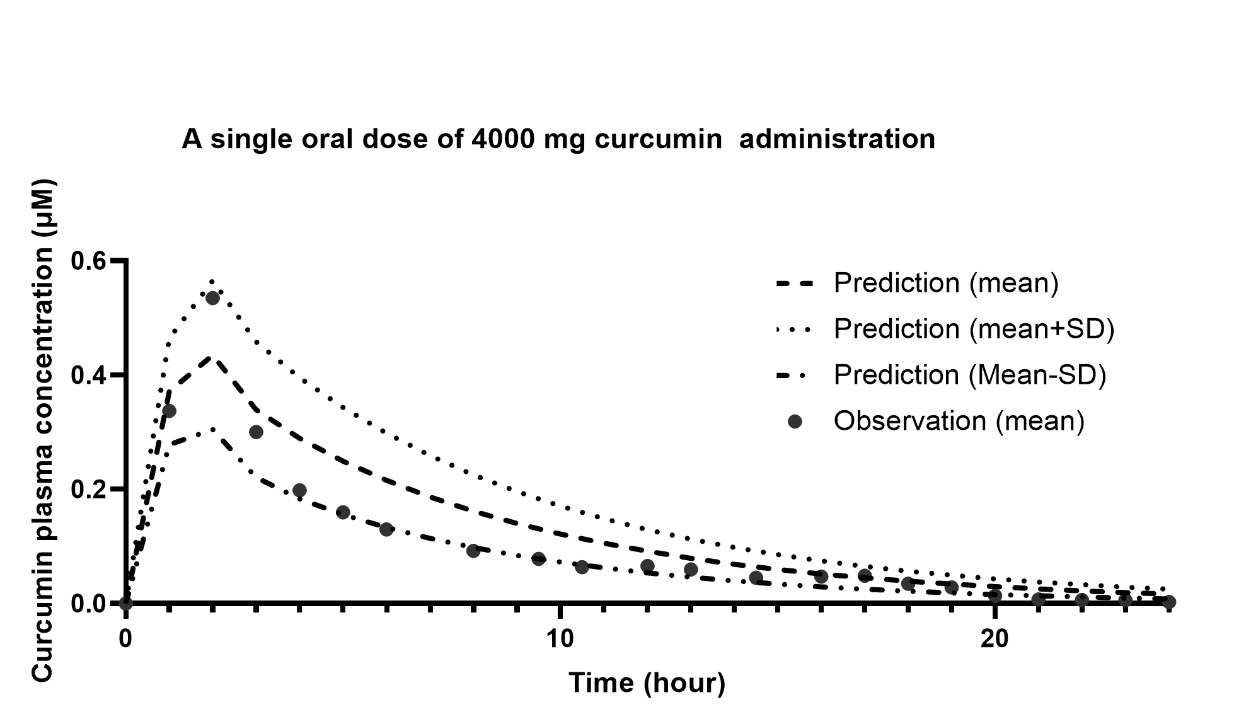


**Figure S2.** A visualization of serum curcumin concentrations between predicted data and observed data at an oral single dose of 6000 mg curcumin administration.


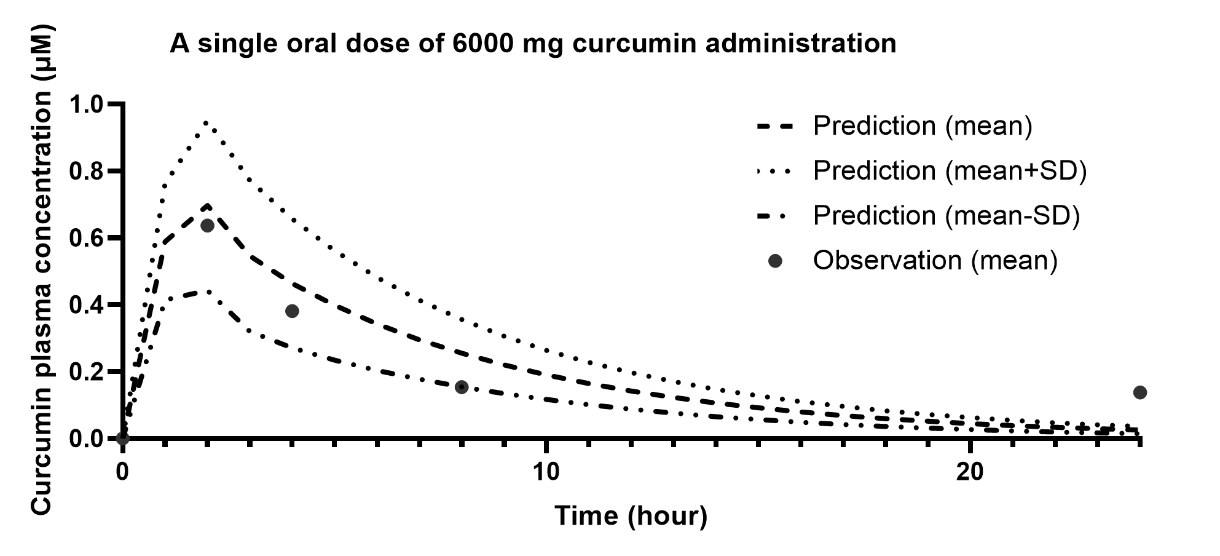


**Figure S3.** A visualization of serum curcumin concentrations between predicted data and observed data at an oral single dose of 8000 mg curcumin administration.


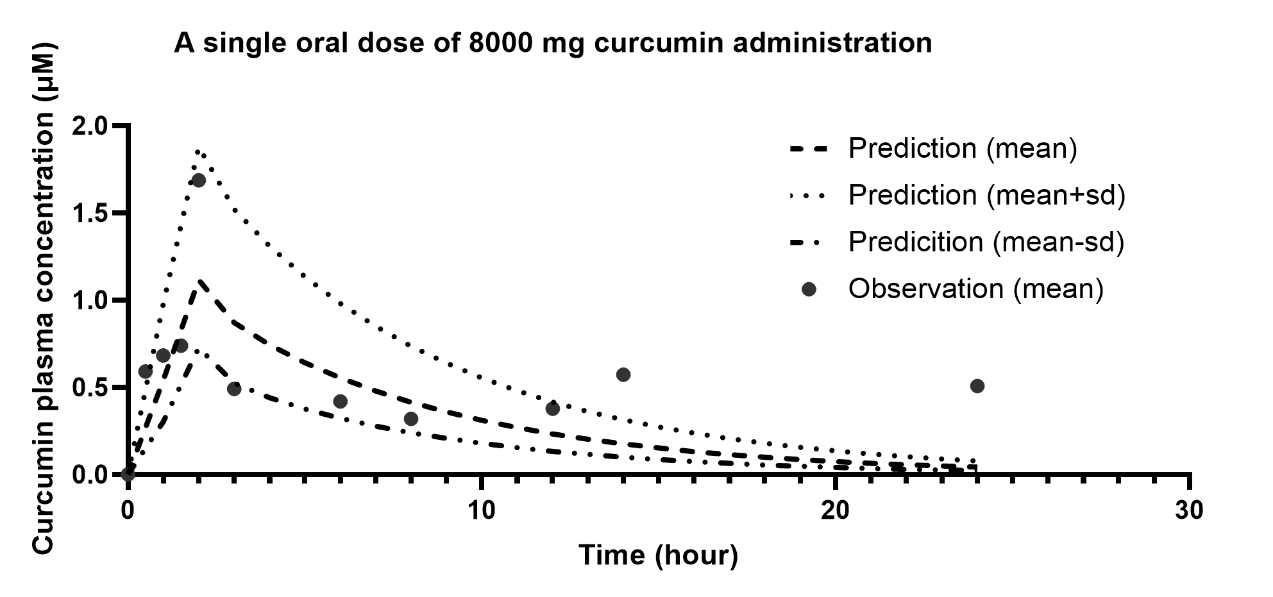


**Supplemental Table 1** Physiologically-based pharmacokinetic (PBPK) model input parameters for chloroquine, lopinavir, ritonavir, and rifampicin.

| Parameter | Curcumin | Lopinavir | Ritonavir | Rifampicin |
| --- | --- | --- | --- | --- |
| Molecular weight (g/ mol) | 368.385 | 628.81 (Wagner et al., 2017) | 721 (Wagner et al., 2017) | 823 (Rasool et al., 2019) |
| Compound type | Polybasic base | Neutral (Wagner et al., 2017) | Dibasic base (Wagner et al., 2017) | Ampholyte (Rasool et al., 2019) |
| Log P | 3.29 | 4.22 (Wagner et al., 2017) | 3.90 (Wagner et al., 2017) | 2.7 (Rasool et al., 2019) |
| pKa | pKa1=7.8, pKa2=8.5, pKa3=9.0 (Hoehle et al., 2007) | NA | 1.8, 2.8 (Wagner et al., 2017) | 1.7, 2.7 (Rasool et al., 2019) |
| R_bp_ (B:P) | 1 (assumed) (Ireson et al., 2002) | 0.75 (Wagner et al., 2017) | 0.58 (Wagner et al., 2017) | 0.67 (Varma et al., 2012) |
| Fraction of unbound drug (fu) | 0.142 (calculated from protein binding) (Privadarsini et al., 2014) | 0.01 (Wagner et al., 2017) | 0.015 (Wagner et al., 2017) | 0.34 (Varma et al., 2012) |
| Absorption rate (1/hours) | NA | 0.74 (Wagner et al., 2017) | 2.31 (Zhang et al., 2012) | 1.15 (Wilkin et al., 2008) |
| Apparent permeability (P_app, A-B_) (10^-6^ cm/s) | 1.13 (Poulin et al., 2002) | 0.0048 (Kigen et al., 2017) | NA | 640 (Stigliani et al., 2006) |
| Apparent permeability (P_app, B-A_) (10^-6^ cm/s) | 1.48 (Poulin et al., 2002) | 0.0320 (Kigen et al., 2017) | NA | 286 (Stigliani et al., 2006) |
| Solubility (mg/L) | 348 (Rahman et al., 2009) | NA | 500 (Xu et al., 2017) | NA |
| In vivo clearance (L/hours) | NA | NA | NA | 10.3 (Seng et al., 2005) |
| Intrinsic clearance (μl/min/mg protein) |  |  |  |  |
| CYP3A4 | NA | 1.4 (Kigen et al., 2017) | 20.14 (Koudriakova et al., 1998) | NA |
| CYP2D6 | NA | NA | 0.93 (Koudriakova et al., 1998) | NA |
| CYP2C8 | NA | NA | NA | NA |
| Hepatic metabolism |  |  |  |  |
| UGT1A1 | 15.6 (Yamazaki et al., 2004) |  |  |  |
| SULT1A1/SULT1A3 | 6.5 (Yamazaki et al., 2004) |  |  |  |
| UGT1A9/UGT1A8/UGT2B7 | 13.4 (Yamazaki et al., 2004) |  |  |  |
| Intestinal metabolism |  |  |  |  |
| UGT1A8/UGT1A10 | 37.5 (Yamazaki et al., 2004) |  |  |  |
| SULT1A1/SULT1A4 | 17.6 (Zeng et al., 2017) |  |  |  |
| UGT1A9/UGT1A8/UGT2B7 | 15.17 (Yamazaki et al., 2004) |  |  |  |
| Renal clearance (L/hours) | NA | NA | NA | 1.5 (Baneyx et al., 2004) |
| Interaction-inhibition |  |  |  |  |
| CYP3A4 | NA | K_inact_=6 (l/h), K_i_=0·257 (mg/L) (Ernest et al., 2005) | K_inact_=19.8 (l/h), K_i_=0.18 (mg/L) (Kirby et al., 2011) | NA |
| Interaction-induction |  |  |  |  |
| CYP3A4 | NA | NA | Ind_max_=2.45, Ind_50_=13.9 (mg/L) (Kirby et al., 2011) | NA |
|  |  |  |  |  |

PBPK: physiologically-based pharmacokinetic, Log P: logarithm of octanol-water partition coefficient, pKa: negative decadal logarithm of acid dissociation constant, B:P: blood-to-plasma partition ratio, K_inact_: inactivation rate of a given enzyme, K_i_: inhibitor concentration yields half-maximal inhibition, Ind_max_: maximal fold induction, Ind_50_ concentration causing 50% maximal induction, P_app, A-B_: apparent permeability from apical to basolateral, P_app, B-A_: apparent permeability from basolateral to apical, NA: not applicable.

**References**

Baneyx, G., Parrott, N., Meille, C., Iliadis, A., Lave, T. (2014). Physiologically based pharmacokinetic modeling of CYP3A4 induction by rifampicin in human: influence of time between substrate and inducer administration. European Journal of Pharmaceutical Sciences, 56, 1-15. <https://doi.org/10.1016/j.ejps2014.02.002>.

Hoehle, S.I., Pfeiffer, E., Metzler, M. (2007). Glucuronidation of curcuminoids by human microsomal and recombinant UDP-glucuronosyltransferases. Molecular Nutrition Food Research, 51, 932-938. <https://doi.org/10.1002/mnfr.200600283>.

Ernest, C.S., Hall, S.D., Jones, D.R. (2005). Mechanism-based inactivation of CYP3A4 by HIV protease inhibitors. Journal of Pharmacology and Experimental Therapeutics, 312, 583-591. <https://doi.org/10.1124/jpet.104.075416>.

Kigen, G., Edwards, G. (2017). BMC Pharmacology & Toxicology, 18, 20. <https://doi.org/10.1186/s40360-017-0129-6>

Kirby,B.J., Collier, A.C., Kharasch, E.D., Whittington, D., Thummel, K.E., Unadkat, J.D. (2011). Complex drug interactions of HIV protease inhibitors 1: inactivation, induction, and inhibition of cytochrome P450 3A by ritonavir or nelfinavir. Drug metabolism and disposition, 39, 1070-1080. <https://doi.org/10.1124/dmd.110.037523>.

Koudriakova, T., Iatsimirskaia, E., Utkin, I., Gangl, E., Vouros, P., Storozhuk, E., et al. (1998). Metabolism of the human immunodeficiency virus protease inhibitors indinavir and ritonavir by human intestinal microsomes and expressed cytochrome P4503A4/3A5: mechanism-based inactivation of cytochrome P4503A by ritonavir. Drug Metabolism & Disposition, 26, 552-561.

Ireson, C.R., Jones, D.J.L., Orr, S., Coughtrie, M.W.H., Boocock, D.J., Williams, M.L., et al. (2002). Metabolism of the cancer chemopreventive agent curcumin in human and rat intestine. Cancer Epidemiology Biomarkers Prevention, 11, 105-1011.

Poulin, P., Theil, F.P. (2002). Prediction of pharmacokinetics prior to in vivo studies. 1. Mechanism-based prediction of volume of distribution. Journal of Pharmaceutical Sciences, 91, 129-156. <https://doi.org/10.1002/jps.10005>.

Priyadarsini, K.I. (2014). The chemistry of curcumin: from extraction to therapeutic agent. Molecules, 19, 20091-112. <https://doi.org/10.3390/molecules191220091>.

Rahman, S.M.H., Telny, T.C., Ravi, T.K., Kuppusamy, S. (2009). Role of surfactant and pH in dissolution of curcumin. Indian Journal of Pharmaceutical Sciences, 71, 139-142. <https://doi.org/10.4103/0250-474X.54280>.

Rasool, M.F., Khalid, S., Majeed, A., Saeed, H., Imran, I., Mohany, M., et al. (2019). Development and evaluation of physiologically based pharmacokinetic drug-disease models for predicting rifampicin exposure in tuberculosis and cirrhosis populations. Pharmaceutics, 11, 578. <https://doi.org/10.3390/pharmaceutics11110578>.

Seng, K.Y., Hee, K.H., Soon, G.H., Chew, N., Khoo, S.H., Lee, L.S.U. (2015). Population pharmacokinetics of rifampicin and 25-deacetyl-rifampicin in healthy Asian adults. Journal of Antimicrobial Agents Chemotherapy, 70, 3298-3306. <https://doi.org/10.1093/jac/dkv268>.

Stigliani, M., Haghi, M., Russo, P., Young, P.M., Traini, D. (2016). Antibiotic transport across bronchial epithelial cells: effects of molecular weight, logP and apparent permeability. European Journal of Pharmaceutical Sciences, 83, 45-51. <https://doi.org/10.1016/j.ejps.2015.12.010>.

Varma, M.V.S., Lai, Y.L., Feng, B., Litchfield, J., Goosen, T.C., Bergman, A. (2012). Physiologically based modeling of pravastatin transporter-mediated hepatobiliary disposition and drug-drug interactions. Pharmaceutical Research, 29, 2860-2873. <https://doi.org/10.1007/s11095-012-0792-7>.

Wagner, C., Zhao, P., Arya, V., Mullick, C., Struble, K., Au, S. (2017). Physiologically based pharmacokinetic modeling for predicting the effect of intrinsic and extrinsic factors on darunavir or lopinavir exposure coadministered with ritonavir. The Journal of Clinical Pharmacology, 57, 1295-1304. <https://doi.org/10.1002/jcph.936>

Wilkins, J.J., Savic, R.M., Karlsson, M.O., Langdon, G., Mclleron, H., Pillai, G., et al. (2008). Population pharmacokinetics of rifampicin in pulmonary tuberculosis patients, including a semimechanistic model to describe variable absorption. Antimicrobial Agents Chemotherapy, 52, 2138-2148. <https://doi.org/10.1128/AAC.00461-07>.

Xu, H., Vela, S., Shi, Y., Marroum, P., Gao, P. (2017). In vitro characterization of ritonavir drug products and correlation to human in vivo performance. Molecular Pharmaceutics, 14, 3801-3814. <https://doi.org/10.1021/acs.molpharmaceut.7b00552>.

Yamazaki, K., Kanaoka, M. (2004). Computational prediction of the plasma protein-binding percent of diverse pharmaceutical compounds. Journal of Pharmaceutical Sciences, 93, 1480-1494. <https://doi.org/10.1002/jps.20059>.

Zeng, Z., Shen, Z.L., Zhai, S., Xu, J.L., Liang, H., Shen, Q., et al. (2017). Transport of curcumin derivatives in Caco-2 cell monolayers. European Journal of Pharmaceutics & Biopharmaceutics, 117, 123-131. <https://doi.org/10.1016/j.ejpb.2017.04.004>.

Zhang, C., Mclleron, H., Ren, Y., van der Walt, J.S., Karlsson, M.O., Simonsson, U., et al. (2012). Population pharmacokinetic of lopinavir and ritonavir in combination with rifampicin-based antitubercular treatment in HIV-infected children. Antiviral Therapy, 17, 25-33. https://doi.org/10.3851/IMP1915.
